# Some Risks of Middle Age

**Published:** 1891-03-07

**Authors:** 


### The Doctor's Armchair.

SOME RISKS OF MIDDLE-AGE.

Middle-aged men and women are not generally the most ornamental, but they are decidedly the most useful portion of the community. The figure may be less graceful, and the movements more ponderous at forty-five; but what the figure has lost in grace the brain has undoubtedly gained in vigour. It is the middle-aged men and women who do the thinking and bear the responsibilities of the world. This is just the period of life when the loss of the father may mean the financial and sometimes the social extinction of the family. The early struggles for a firm foothold in business or professional life are more or less past, and prosperity is well in view. It is within view, and has perhaps been touched, or even laid hold of; but it has not been so long held in possession as to have made the family fortunes quite secure. Ten or fifteen years longer are generally required before that is accomplished. The father now walks the deck of his own ship, and if he can but keep himself alive and in health for another dozen years, the whole family may be brought into the haven of assured prosperity and comfort.

It is clear that the most important member of the household at this period is the father; and it is equally clear that the most important thing about the father is his health. By health here is meant bodily vigour, and also, as a matter of course, mental capacity and moral equilibrium. All the three must be insisted upon as necessary components of true health. A deficiency in mental capacity, or an instability in moral behaviour are as much aberrations from scientific health as indigestion, typhoid fever, or a disordered nervous system. We have come to a time now, in this country, when it is clearly perceived by intelligent minds that a moral twist or a degree of mental incapacity is as truly a departure from the healthful standard as any ailment which is obviously physical. It is important to say these things, even though the man who says them may be conscious that some of his readers will think he is leaving the chair of the doctor of the body and ascending the pulpit of the doctor of the soul. Nothing of the kind. The surest method of gaining bodily health is to use the reason rightly and to conform to the highest moral standard. A defect in morals is evidence of a defect in intellect, and a defect in intellect is equivalent to the absence of one of the principal safeguards that protect the bodily health.

There are certain dangers to which middle-aged men are exposed, and which bring about a definite loss of life every year. The writer, for example, knows of two cases, not in his own practice, of death from double-pneumonia during the last four weeks. The patients were men of capacity and energy, and both were successful in business. The age of the one was forty-four; that of the other forty-six. The younger was at his office on a given Wednesday, but lay dead in his bed on the following Monday. The elder was at his warehouse on a given Thursday afternoon, and he also was dead on the following Monday week. Both of these men were in the full vigour of health and in the very prime of manhood. Their habits were excellent and their prospects of the best. Each of them should have lived to be eighty; and certainly neither of them would have died at the present time of pneumonia if they had understood the art of managing their health, and had practised what they understood.

The first thing for a town man to understand is, that he is a town man. The young man who comes up fresh from the country at twenty, may be likened to a boy in a circus who is walking on the solid ground. The same young man, after he has been in London twenty years, may be likened to a man in a circus who is walking on a tight-rope forty feet from the ground. The health of the young man at twenty, fresh from the country, cannot easily be upset, and when it is upset, it has not usually any further to fall than has the body of the youth who is walking on terra firma in the circus, if he should happen to stumble. But the health of the town man at forty is as delicately balanced as the body of the man on the tight rope. The least uncertain movement either way, and down it goes; aad it may plunge through forty or even through a hundred feet of space, down to a dangerous typhoid fever, or to a fatal double-pneumonia.

"What is a business man to do, if this be truth"? That is the anxious question which the father of a young family and the head of a business firm feels driven to ask. Do! Take the trouble to understand yourself first of all, and then to understand the surroundings amidst which you live! You understand your banking account; you know how much it will bear, and how much it will not bear; you know there is a point beyond which its utmost elasticity cannot be made to stretch. But your health is the very keystone on which the structure, not only of your banking account, but of every other part of your social, financial, and family well-being rests. Take away the keystone, and the whole building tumbles to the ground. Ninety-nine readers in every hundred will believe these statements when they see them; not more than one in every hundred will think of making any use of them in practice. Now, the doctor has a very simple explanation of such peculiar conduct. He has arrived at this conclusion, after much experience and many reasonings, namely: That any man who sees a truth clearly, and knows that the truth contains a commandment, is a fool if he does not obey it. At bottom, the greater part of our want of health and our want of happiness is due to our want of mental capacity.

Here are two or three examples of the kinds of danger that are part of a town man's environment. The morning and evening railway trains kill hundreds of people every winter. A man, for instance, has had a bad cold, and his chest is at the "raw" stage. He has a ten-mile journey to town every morning. He sits down with his face to the engine, and opens the window. In rushes the fog-laden wind of November, or the still more piercing and deadly blast of March. The three-quarters of an hour between his home and the City are just sufficient for pleurisy, pneumonia, bronchitis, or rheumatic fever to lay a fatal hand upon him. What is he to do? Close the window and keep out the draught at all costs! But does he then not run the risk of being poisoned by bad air? The injury which can be got from a bad atmosphere in the worst compartment of the worst railway carriage that was ever constructed is as nothing compared with the deadly dangers that lurk in a concentrated inrush of winter air travelling at the rate of twenty or thirty miles an hour. Another example of an everyday danger is the great irregularity of the town man's meals and the too long intervals between them. An ordinary business man should have a sufficient meal every four hours; and he should make it a part of his duty to be as punctual as the clock. Many more details of danger to middle life might be given. From the examples here presented the reader is asked to discover others for himself. Let him, however, remember this, that in the judgment of the medical profession, the health of the average town man is in as unstable a condition, and requires to be as nicely balanced as the body of the tight-rope dancer. Nothing but an intelligent understanding of his own state and surroundings, and a reasonable obedience to the dictates of physiological sense can save the ordinary town-dweller from dying more or fewer years before his time.

